# Urinary excretion of uric acid is negatively associated with albuminuria in patients with chronic kidney disease: a cross-sectional study

**DOI:** 10.1186/s12882-018-0892-7

**Published:** 2018-04-24

**Authors:** Fengqin Li, Hui Guo, Jianan Zou, Weijun Chen, Yijun Lu, Xiaoli Zhang, Chensheng Fu, Jing Xiao, Zhibin Ye

**Affiliations:** 1 Department of Nephrology, Huadong Hospital affiliated to Fudan University, No. 221 West Yan’an Road, Shanghai, 200040 People’s Republic of China; 2 Shanghai Key Laboratory of Clinical Geriatric Medicine, No. 221 West Yan’an Road, Shanghai, 200040 People’s Republic of China

**Keywords:** Albuminuria, 24 h urinary uric acid excretion, Fractional excretion of uric acid, Uric acid clearance rate, Chronic kidney disease

## Abstract

**Background:**

Increasing evidence has shown that albuminuria is related to serum uric acid. Little is known about whether this association may be interrelated via renal handling of uric acid. Therefore, we aim to study urinary uric acid excretion and its association with albuminuria in patients with chronic kidney disease (CKD).

**Methods:**

A cross-sectional study of 200 Chinese CKD patients recruited from department of nephrology of Huadong hospital was conducted. Levels of 24 h urinary excretion of uric acid (24-h Uur), fractional excretion of uric acid (FEur) and uric acid clearance rate (Cur) according to gender, CKD stages, hypertension and albuminuria status were compared by a multivariate analysis. Pearson and Spearman correlation and multiple regression analyses were used to study the correlation of 24-h Uur, FEur and Cur with urinary albumin to creatinine ratio (UACR).

**Results:**

The multivariate analysis showed that 24-h Uur and Cur were lower and FEur was higher in the hypertension group, stage 3–5 CKD and macro-albuminuria group (UACR> 30 mg/mmol) than those in the normotensive group, stage 1 CKD group and the normo-albuminuria group (UACR< 3 mg/mmol) (all *P* < 0.05). Moreover, males had higher 24-h Uur and lower FEur than females (both *P* < 0.05). Multiple linear regression analysis showed that UACR was negatively associated with 24-h Uur and Cur (*P* = 0.021, *P* = 0.007, respectively), but not with FEur (*P* = 0.759), after adjusting for multiple confounding factors.

**Conclusions:**

Our findings suggested that urinary excretion of uric acid is negatively associated with albuminuria in patients with CKD. This phenomenon may help to explain the association between albuminuria and serum uric acid.

## Background

Uric acid homeostasis is determined by the balance between production, intestinal secretion, and renal excretion [1]. The kidney is an important regulator of circulating uric acid levels. Approximately two-thirds of uric acid is excreted by the kidneys, while the remaining one-third is excreted by intestinal uricolysis [2]. The renal proximal tubule is responsible for almost all renal urate transport and is where urate re-absorption primarily occurs [3]. Proximal renal tubular epithelial cells (PTECs) excrete urate and express ion and urate transport channels [1]. It was recognized that defective renal handling of uric acid accounted for 90% of the incidence of hyperuricemia and gout, including reduced glomerular filtration rate or enhanced reabsorption or insufficient secretion of renal tubules [4–6]. Previous researchers studied urinary excretion of uric acid in gouty patients, diabetes, hypertension and general population. Although many studies have shown that hyperuricemia is common in chronic kidney disease (CKD) and associated with the development and progression of CKD [7–9], urinary uric acid excretion in population with CKD is rarely studied. Early studies indicated that residual nephrons of the patients with CKD underwent a progressive increase in fractional excretion of uric acid as nephron number decreases. Until advanced uremia supervened, residual nephrons of the CKD patients retained the integrity of uric acid reabsorption and secretory transport [10, 11]. Moreover, the extra renal uric acid excretion assumed a great role in CKD and eventually became the major route of elimination of uric acid [12]. In fact, with aging of population and improvement of the life quality, the incidence of CKD is rising rapidly, accompanied by metabolic abnormalities [13]. However, what factors may be involved in renal uric acid excretion are not yet fully understood.

Albumin lost in urine is the result of the amount filtered by the glomerulus and the amount that escapes degradation or reabsorption by the proximal tubule, and it is a sign of early renal damage and a definite risk factor for the development of CKD [14]. Over the last decades, several studies have shown an association between albuminuria and SUA, observed in subjects with heart failure, diabetes mellitus, hypertension, and general population [15–19]. Interestingly, recent studies have documented that proximal epithelial tubular cells that are exposed to albumin up- and down-regulated genes encoding for membrane transporters differentially [20–22]. Tudpor et al. found that plasmin in the urinary of patients with proteinuria influenced the activity of membrane transporters of tubular epithelial cells [23]. Given these data, we questioned whether the association between albuminuria and serum uric acid may be interrelated via renal handling of uric acid. Therefore, we aimed to study the influencing factors of urinary uric acid excretion, especially the association of albuminuria with urinary uric acid excretion in CKD patients.

## Methods

### Subjects

A total of 200 patients (93 males/107 females) with a mean age of 53.5 ± 17.2 (range18–89) years with CKD hospitalized at Huadong hospital affiliated to Fudan University (Shanghai, P.R.China) between July 10, 2015 and June 10, 2017 were admitted for analysis in this study. All patients were clinically stable during the study period. Those who had taken drugs that could affect uric acid metabolism within the previous 2 weeks, such as diuretic, aspirin, cyclosporine, glucocorticoids, sodium bicarbonate, levodopa, immunosuppressive agents, anti-tuberculosis drugs, losartan, metformin, fenofibrate, uric acid-lowering agents, such as allopurinol, febuxostat or benzbromarone were excluded from this study. Excluded also subjects with a history of hereditary hyperuricemia, kidney transplantation, dialysis, infection, severe heart, lung or liver dysfunction, tumor and hematologic diseases, hyperparathyroidism and shock. Moreover, all patients avoided high purine and fructose rich diet and alcohol for 5 days before the study.

Our study was approved by the ethical review board of Huadong hospital affiliated to Fudan University, and our study conformed to the ethics guidelines. Written informed consents were obtained from all subjects.

### Clinical and laboratory measurements

Age and gender were recorded. Weight, height and blood pressure were measured by trained nurses in a standardized way. Body mass index (BMI) was calculated as weight in kilograms divided by height in meters squared. To obtain information on the underlying cause of CKD, the patient’s treating nephrologists were asked to choose from a given list of etiologic categories, which were summarized into the groups: primary glomerulonephritis (PGN), diabetic kidney disease (DKD), hypertensive nephropathy(HN), tubulointerstitial nephropathy (TIN), secondary glomerulonephritis (SGN), polycystic kidney disease (PKD) and atherosclerotic renal arterial stenosis (ARAS). Morning blood samples were obtained after 12 h of fasting on the day of collecting 24 h of urine and were subsequently analyzed in the laboratory at Huadong hospital. The serum levels of uric acid (SUA), serum creatinine (Scr), blood urea nitrogen (BUN), C-reactive protein (CRP), total cholesterol (TC), triglycerides (TG), high-density lipoprotein (HDL), low-density lipoprotein (LDL), fasting blood glucose (FBG) and hemoglobin A1c (HbA1c) were measured. The estimated glomerular filtration rate (eGFR) (milliliters per minute per 1.73 m^2^), an indicator of renal function, was calculated using the Chronic Kidney Disease Epidemiology Collaboration (CKD-EPI) formula [24].

The participants were given a three-liter plastic bottle containing acid preservatives and standardized written instructions on 24-h urine collection [25]. A single 24-h urinary specimen was collected. 24-h urinary levels of uric acid (Uur), urinary creatinine (Ucr), urinary volume (UV), urinary pH (UpH), urinary albumin(UALB), urinary β_2_ microglobulin and urinary glucose (Ug) were measured. Fractional excretion of uric acid (FEur) was calculated as (Uur × Scr)/(SUA × Ucr) × 100, expressed as percentage. Uric acid clearance rate (Cur) was calculated as Uur × UV/SUA. The urinary albumin to creatinine ratio (UACR) was based on UALB/Ucr. Urinary β_2_ microglobulin-creatinine ratio (UBCR) was calculated as urinary β_2_ microglobulin / Ucr. Twenty-four-hour Uur, Ug and Cur were corrected for body surface area.

### Diagnostic and grouping criteria

According to the WHO organization, we divided patients into Y1: age < 45 years, the youth; Y2: 45 ≤ age ≤ 65 years, the middle-age; Y3: age > 65 years, the elderly; hyperuricemia was defined as the serum level of uric acid > 420 μmol/L; Hypertension was defined as systolic blood pressure (SBP) ≥ 140 mmHg, diastolic blood pressure (DBP) ≥90 mmHg, or use of antihypertensive medication according to self-report or to pharmacy data. According to the classification of hypertension, the patients were divided into H0 (SBP < 140 mmHg, DBP < 90 mmHg), H1 (140 ≤ SBP ≤ 159 mmHg, 90 ≤ DBP ≤ 99 mmHg), H2 (160 ≤ SBP ≤ 179 mmHg, 100 ≤ DBP ≤ 109 mmHg), H3 (SBP ≥ 180 mmHg, DBP ≥ 110 mmHg); Diabetes was defined as a fasting glucose level of > 7.0 mmol/L, a non-fasting glucose level of > 11.1 mmol/L or use of anti-diabetic medication according to self-report or medical history. And all diabetic patients in this study were diagnosed with type 2 diabetes. The diabetes patients were divided into D0 (non-diabetic group), D1 (pre-diabetes group) and D2 (diabetes group). Pre-diabetes included both impaired fasting glucose (IFG) and impaired glucose tolerance (IGT); According to the Kidney Disease: Improving Global Outcomes CKD guidelines, GFR categories were defined as follows: ≥90 mL/min/1.73m^2^ (CKD1), 60–89 mL/min/1.73m^2^ (CKD2), 30–59 mL/min/1.73m^2^ (CKD3), 15–29 mL/min/1.73m^2^ (CKD4) and < 15 mL/min/1.73m^2^ (CKD5); Albuminuria was defined as UACR≥3 mg/mmol. UACR was categorized into A1(UACR< 3 mg/mmol, normo-albuminuria), A2 (3 ≤ UACR≤30 mg/mmol, microalbuminuria) and A3 (UACR> 30 mg/mmol, macro-albuminuria) [24]; The index of renal uric acid excretion includes 24-h Uur, FEur and Cur. Inefficient renal excretion of uric acid was defined as 24-h Uur < 3.6 mmol/24 h/1.73m^2^. According to tertiles of 24-h Uur, patients were divided into three groups (U0: 24-h Uur < 2.0 mmol/24 h/1.73m^2^; U1: 2.0 ≤ 24-h Uur ≤ 2.9 mmol/24 h/1.73m^2^; U2: 24-h Uur > 2.9 mmol/24 h/1.73m^2^).

### Statistical analysis

The continuous and categorical clinical variables are reported in means±SD and percentages, respectively. In case of nonparametric data distribution medians with inter quartile range (IQR) are presented. T test was used between the two groups, and the univariate analysis of variance (ANOVA) was used to measure the data among the groups or a Kruskal-Wallis test in case of nonparametric data distribution. Differences between groups for proportions were tested with a chi-square test. Before conducting the multiple correlation analysis, we firstly verified that independent variables were not correlated among them. We analyzed the multiple collinearity by calculating the correlation coefficient matrix, tolerance and variance inflation factor of independent variables. The analysis showed that the correlation coefficient of any two independent variables was less than 0.6, all the values of tolerance were greater than 0.1 (the minimum value is 0.413), and all the values of variance inflation factor were less than 10 (the maximum value is 2.419), which suggested that there was no multiple collinearity. We then used multivariate analysis to test the influence of each factor (e.g. sex, age, CKD stage, hypertension stage, diabetes stage, UACR and original pathology etc) on separate variable and their interaction. When the multivariate analysis is significant and no interaction is found, we performed further post-hoc test to determine the difference between two groups according to these statistically significant factors. As for the post-hoc test, we used least significance difference (LSD) test if the variance was homogeneous, and we used Tamhane’s T2 test if not. Pearson and Spearman correlation analysis were used to find the risk factors associated with urinary uric acid excretion. Since UACR, UBCR, FEur, TG, CRP and Ug were not normally distributed, we performed Spearman correlation analysis about these variables. Multiple linear regression analyses were then performed to determine the association of albuminuria with renal uric acid excretion. Statistical significance for all analyses was set at *P* < 0.05. Statistical analysis was performed with software SPSS 22.0.

## Results

### Characteristics of the study population and when stratified by tertiles of 24-h Uur

We included 200 participants with mean age 53.5 ± 17.2 years, and 93 males (46.5%). General data are shown in Table 1. Hyperuricemia was present in 45 (22.5%) patients, and inefficient renal excretion of uric acid (24-h Uur < 3.6 mmol/24 h/1.73m^2^) was present in 91.3% (42/46) among them. Sixty-seven participants had microalbuminuria (3 ≤ UACR≤30 mg/mmol) and 88 participants had macro-albuminuria (UACR> 30 mg/mmol). Calcium channel blockers, β blockers, statins, α-glucosidase inhibitor or insulin were used by the patients with CKD, and these were similar among groups. The comparison of clinical and laboratory indexes among groups U0 (24-hUur< 2.0 mmol/24 h/1.73m^2^), U1 (2.0 ≤ 24-hUur≤2.9 mmol/24 h/1.73m^2^) and U2 (24-hUur> 2.9 mmol/24 h/1.73m^2^) is also included in Table 1. Gender, age, BMI, eGFR, SUA, UpH, UACR, UBCR, UV, FEur, Cur and the proportion of hypertension and hyperuricemia significantly differed in three groups (all *P* < 0.05, Table 1).

**Table 1:** Characteristics of the study population stratified by tertiles of 24-h Uur

Variables	All (*n* = 200)	U0 (*n* = 63)	U1 (*n* = 74)	U2 (n = 63)	*P* value
		< 2.0 mmol/24 h/1.73m^2^	2.0–2.9 mmol/24 h/1.73m^2^	> 2.9 mmol/24 h/1.73m^2^	
Male gender (%)	46.5	33.3	50.0*	55.6*	0.033
Age (years)	53.5 ± 17.2	58.7 ± 16.9	56.6 ± 16.8	44.5 ± 14.3**	< 0.001
BMI (kg/m^2^)	23.9 ± 3.8	22.3 ± 2.9	24.0 ± 3.8*	25.4 ± 3.9**	< 0.001
eGFR (ml/min/1.73m^2^)	65.2 ± 39.3	40.5 ± 36.8	66.4 ± 34.8**	88.8 ± 31.5**	< 0.001
SUA (umol/L)	363.6 ± 102.0	399.0 ± 123.7	364.9 ± 91.7	326.6 ± 74.1**	< 0.001
TC (mmol/L)	4.8 ± 1.3	4.8 ± 1.0	4.8 ± 1.1	4.9 ± 1.6	0.865
TG (mmol/L)	1.5 (1.0, 2.2)	1.4 (1.0, 2.1)	1.5 (1.1, 2.5)	1.5 (1.0, 2.2)	0.599
HDL (mmol/L)	1.3 ± 0.3	1.3 ± 0.3	1.3 ± 0.4	1.3 ± 0.3	0.516
LDL (mmol/L)	2.8 ± 0.9	2.8 ± 0.8	2.9 ± 0.9	2.8 ± 1.0	0.903
HbA1c (%)	6.0 ± 1.1	5.8 ± 0.9	6.3 ± 1.4	6.0 ± 0.9	0.097
FBG (mmol/L)	5.1 ± 1.5	4.8 ± 1.3	5.2 ± 1.9	5.2 ± 1.0	0.205
CRP (mg/L)	2.5 (0.8, 4.9)	1.8 (0.8, 4.3)	2.6 (0.8, 6.1)	2.8 (0.8, 4.9)	0.508
UpH	6.2 ± 0.6	6.4 ± 0.7	6.1 ± 0.5*	6.2 ± 0.5	0.001
UV (ml/24 h)	1850.9 ± 691.1	1609.2 ± 569.6	1895.4 ± 598.2*	2040.2 ± 830.0**	0.001
Ug (mmol/24 h/1.73m^2^)	0.6 (0.2, 2.0)	0.4 (0.2, 4.8)	0.5 (0.3, 1.4)	0.8 (0.3, 1.8)	0.465
UACR (mg/mmol)	24.6 (3.9, 131.8)	61.3 (15.8, 219.5)	26.3 (3.9, 126.8)	9.6 (2.0, 50.7) *	0.002
UBCR (mg/mmol)	0.08 (0.0, 0.5)	0.2 (0.0, 3.2)	0.1 (0.0, 0.5) **	0.1 (0.0, 0.1) **	< 0.001
Hypertension (%)	60.0	71.4	64.9	42.9*	0.003
Diabetes (%)	28.0	27.0	29.7	27.0	0.917
Hyperuricemia (%)	22.5	36.5.	21.6	9.5**	0.001
FEur (%)	7.4 (5.8, 11.6)	9.6 (6.2, 16.8)	7.3 (5.5, 10.4)	6.8 (5.6, 9.8) *	0.008
Cur (ml/min/1.73m^2^)	5.5 ± 3.3	3.0 ± 1.9	5.0 ± 1.6**	8.6 ± 2.6**	< 0.001

*Abbreviations*: *BMI* body mass index, *eGFR* estimated glomerular filtration rate, *CRP* C-reactive protein, *TC* total cholesterol, *TG* triglycerides, *HDL* high-density lipoprotein, *LDL* low-density lipoprotein, *HbA1c* hemoglobin A1c, *FBG* fasting blood glucose, *SUA* serum uric acid, *Ug* urinary glucose, *UV* urinary volume, *UpH* urinary pH, *UACR* urinary albumin to creatinine ratio, *UBCR* urinary β_2_ microglobulin-creatinine ratio, *24-h Uur* 24-h urinary uric acid excretion, *FEur* fractional excretion of uric acid, *Cur* uric acid clearance rate

*P < 0.05, **P < 0.01 vs. the U0. Using the least significance difference (LSD) method if the variance is equal or the Tamhane’s T2 method if the variance is not equal, using Kruskal-Wallis test in case of nonparametric data distribution. Numbers are mean and SD or proportion. For TG, UACR, UBCR, CRP, Ug and FEur, median and the 25th and 75th percentile are shown

Among the etiology of CKD, PGN accounted for the highest proportion (56.5%, 113/200), followed by DKD (14.5%, 29/200). In addition, HN, TIN, SGN, PKD and ARAS accounted for 12.5% (25/200), 6% (12/200), 4% (8/200), 3.5% (7/200) and 3% (6/200), respectively.

### Differences in urinary uric acid excretion according to gender, CKD, hypertension, and albuminuria status

According to the multivariate analysis, there was statistical significance in the influence of CKD stage, hypertension stage, UACR on 24-h Uur, Cur and FEur, and statistical significance in the influence of sex on 24-h Uur and FEur. There was no statistical significance in the influence of age, diabetes stage, and the original etiology on 24-h Uur, Cur and FEur, and no statistical significance in the influence of sex on Cur.

**Fig. 1:**
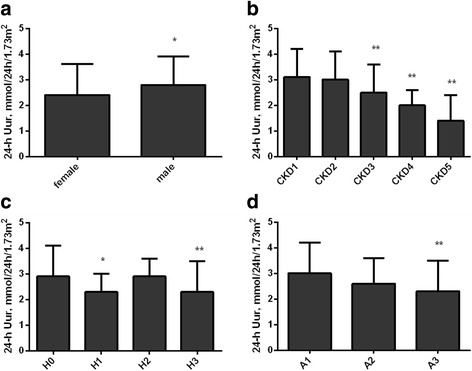
Levels of 24-hUur according to gender, CKD, hypertension, and albuminuria status. Levels of 24-h Uur were compared among different groups in gender (**a**), CKD stages (**b**), hypertension status (**c**), and albuminuria status (**d**) using a multivariate analysis. Males had higher 24-h Uur than females (**a**). Compared with stage 1 CKD group, 24-h Uur decreased in stage 3–5 CKD groups (**b**). The level of 24-h Uur was lower in stage 1 and 3 hypertension group than that in normo-tension group (**c**). Furthermore, participants in the macro-albuminuria group had significantly lower 24-h Uur compared with those in the normo-albuminuria group (**d**). **a** 24-h Uur in female and male; (**b**) 24-h Uur in CKD stage1–5; (**c**) 24-h Uur in H0: normo-tension; H1:stage 1 hypertension; H2: stage 2 hypertension; H3: stage 3 hypertension; (**d**) 24-h Uur in A1: UACR< 3 mg/mmol, normo-albuminuria; A2: 3 ≤ UACR≤30 mg/mmol, microalbuminuria; A3: UACR> 30 mg/mmol, macro-albuminuria. Error bars represent the standard deviation values. * *P* < 0.05, ** *P* < 0.01 vs. female, CKD1, H0, A1 respectively. Abbreviations: 24-h Uur, 24-h urinary uric acid excretion; UACR, urinary albumin to creatinine ratio; CKD, chronic kidney disease

As to 24-h Uur, males had higher 24-h Uur than females (*P* < 0.05; Fig. 1a). Compared with stage 1 CKD group, 24-h Uur decreased in stage 3–5 CKD groups (all *P* < 0.01; Fig. 1b). And 24-h Uur was lower in stage 1 and 3 hypertension groups than that in normo-tension group (*P* < 0.05, *P* < 0.01; Fig. 1c). Furthermore, participants in the macro-albuminuria group had significantly lower 24-h Uur compared with those in the normo-albuminuria group (*P* < 0.01; Fig. 1d).

**Fig. 2:**
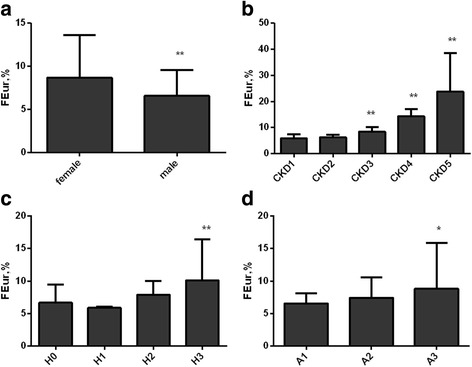
Levels of FEur according to gender, CKD, hypertension, and albuminuria status. Levels of FEur were compared among different groups in gender (**a**), CKD stages (**b**), hypertension status (**c**), and albuminuria (**d**) using a multivariate analysis. Females had higher levels in FEur than males (**a**). Compared with stage 1 CKD group, FEur increased in stage 3–5 CKD groups (**b**). And FEur was higher in stage 3 hypertension group than that in normo-tension group (**c**). Furthermore, participants in the macro-albuminuria group had significantly higher FEur compared with those in the normo-albuminuria group (**d**). **a** FEur in female and male; (**b**) FEur in CKD 1–5 stage; (**c**) FEur in H0: normo-tension; H1: stage 1 hypertension; H2: stage 2 hypertension; H3: stage 3 hypertension; (**d**) FEur in A1: UACR< 3 mg/mmol, normo-albuminuria; A2: 3 ≤ UACR≤30 mg/mmol, microalbuminuria; A3: UACR> 30 mg/mmol, macro-albuminuria. Error bars represent the standard deviation values. Error bars represent the interquartile range (IQR). *P < 0.05, ** *P* < 0.01 vs. female, CKD1, H0, A1 respectively. Abbreviations: FEur, fractional excretion of uric acid; UACR, urinary albumin to creatinine ratio; CKD, chronic kidney disease

As for FEur, females had higher levels of FEur than males (*P* < 0.01; Fig. 2a). Compared with stage1 CKD group, FEur increased in stage 3–5 CKD groups (all *P* < 0.01; Fig. 2b). And FEur was higher in stage 3 hypertension group than that in normo-tension group (*P* < 0.01; Fig. 2c). Furthermore, participants in the macro-albuminuria group had significantly higher FEur compared with those in the normo-albuminuria group (*P* < 0.05; Fig. 2d).

**Fig. 3:**
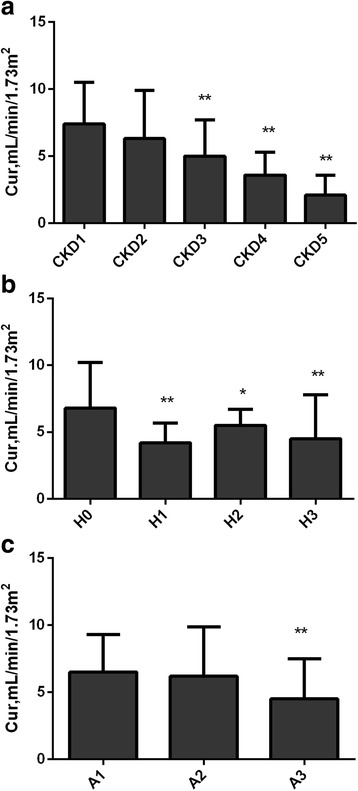
Levels of Cur according to CKD, hypertension, and albuminuria status. Levels of Cur were compared among different groups in CKD stages (**a**), hypertension status (**b**), and albuminuria status (**c**) using a multivariate analysis. Compared with stage 1 CKD, Cur decreased in stage 3–5 CKD (**a**). The level of Cur was lower in stage 1–3 hypertension than that in normo-tension group (**b**). Furthermore, participants in the macro-albuminuria group had significantly lower Cur compared with those in the normo-albuminuria group (**c**). **a** Cur in CKD stage1–5; (**b**) Cur in H0: normo-tension; H1: stage 1 hypertension; H2: stage 2 hypertension; H3: stage 3 hypertension; (**c**) Cur in A1: UACR< 3 mg/mmol, normo-albuminuria; A2: 3 ≤ UACR≤30 mg/mmol, microalbuminuria; A3: UACR> 30 mg/mmol, macro-albuminuria. Error bars represent the standard deviation values. * *P* < 0.05, ** *P* < 0.01 vs. CKD1, H0, A1 respectively. Abbreviations: Cur, uric acid clearance rate; UACR, urinary albumin to creatinine ratio; CKD, chronic kidney disease

With regard to Cur, compared with stage 1 CKD group, Cur decreased in stage 3–5 CKD groups (all *P* < 0.01; Fig. 3a). And Cur was lower in stage 1–3 hypertension groups than that in normo-tension group (all *P* < 0.05; Fig. 3b). Furthermore, participants in the macro-albuminuria group had significantly lower Cur compared with those in the normo-albuminuria group (*P* < 0.01; Fig. 3c).

### Relationship between albuminuria and urinary uric acid excretion

Pearson and Spearman correlation analysis showed that 24-h Uur was positively associated with eGFR (*r* = 0.449, *P* < 0.001), BMI (*r* = 0.321, *P* < 0.001) and UV (*r* = 0.292, *P* < 0.001). Twenty four-hour Uur was negatively associated with age (*r* = − 0.322, *P* < 0.001), UpH (*r* = − 0.214, *P* < 0.001), SUA (*r* = − 0.183, *P* = 0.009) and UBCR (*r* = − 0.348, *P* < 0.001). FEur was positively associated with age (*r* = 0.282, *P* < 0.001), CRP (*r* = 0.187, *P* = 0.008), UpH (*r* = 0.311, *P* < 0.001), Ug (*r* = 0.208, *P* = 0.003) and UBCR (*r* = 0.628, *P* < 0.001). FEur was negatively associated with BMI (*r* = − 0.207, *P* = 0.003) and eGFR (*r* = − 0.644, *P* < 0.001). Cur was positively associated with UV (*r* = 0.301, *P* < 0.001) and eGFR (*r* = 0.519, *P* < 0.001). Cur was negatively associated with age (*r* = − 0.298, *P* < 0.001), SUA (*r* = − 0.619, *P* < 0.001), UpH (*r* = − 0.178, *P* = 0.012) and UBCR (*r* = − 0.306, *P* < 0.001). These factors were recognized as confounding factors for albuminuria on urinary uric acid excretion.

Spearman correlation coefficient demonstrated that 24-h Uur was negatively associated with UACR (*r* = − 0.263, *P* < 0.001) before adjusting for the confounding factors. After adjusting for confounding factors in three models (model 1 adjusted for sex, age and BMI; model 2 plus eGFR and SUA; model 3 plus CRP, UpH, TG, UBCR, UV and Ug), the association of 24-h Uur with UACR was still statistically significant (*P* = 0.021; Table 2).

**Table 2:** Multiple linear regression analysis for association of UACR (independent variable) with 24-h Uur (dependent variable)

	Model 1		Model 2		Model 3	
	*St B*	*P*	*St B*	*P*	*St B*	*P*
UACR, mg/mmol	−0.218	< 0.001	− 0.147	0.015	− 0.140	0.021
Gender (female vs male)	0.158	0.011	0.158	0.009	0.168	0.005
Age, y	−0.336	< 0.001	− 0.213	0.001	− 0.158	0.016
BMI, kg/ m^2^	0.292	< 0.001	0.288	< 0.001	0.212	< 0.001
eGFR, ml/min//1.73m^2^			0.288	< 0.001	0.237	0.006
TG, mmol/L					0.169	0.005
UV, ml/24 h					0.224	< 0.001

*Abbreviations*: *UACR* urinary albumin to creatinine ratio, *24-h Uur* 24 h urinary uric acid excretion, *BMI* body mass index, *eGFR* estimated glomerular filtration rate, *SUA* serum uric acid, *CRP* C-reactive protein, *UpH* urinary pH, *TG* triglycerides, *UBCR* urinary β_2_ microglobulin-creatinine ratio, *UV* urinary volume, *Ug* urinary glucose

model 1 adjusted for sex, age and BMI; model 2 plus eGFR and SUA; model 3 plus CRP, UpH, TG, UBCR, UV and Ug. Standardized beta coefficients (*St B*) refer to how many standard deviations a dependent variable will change per standard deviation increase in the predictor variable

FEur was positively associated with UACR (*r* = 0.247, *P* < 0.001) with a Spearman correlation coefficient before adjusting for the confounding factors. After adjusting for confounding factors in three models (model 1 adjusted for sex, age and BMI; model 2 plus eGFR and SUA; model 3 plus CRP, UpH, TG, UBCR, UV and Ug), the correlations of UACR with FEur disappeared (*P* > 0.05; Table 3).

**Table 3:** Multiple linear regression analysis for the association of UACR (independent variable) with FEur (dependent variables)

	Model 1		Model 2		Model 3	
	*St B*	*P*	*St B*	*P*	*St B*	*P*
UACR, mg/mmol	0.203	0.003	0.118	0.051	0.016	0.759
eGFR, ml/min/1.73m^2^			−0.659	< 0.001	−0.386	< 0.001
SUA,umol/L			−0.322	< 0.001	−0.253	< 0.001
UBCR, mg/mmol					0.518	< 0.001
CRP, mg/L					0.154	0.004

*Abbreviations*: *UACR* urinary albumin to creatinine ratio, *FEur* fractional excretion of uric acid, *BMI* body mass index, *eGFR* estimated glomerular filtration rate, *SUA* serum uric acid, *CRP* C-reactive protein, *UpH* urinary pH, *TG* triglycerides, *UBCR* urinary β_2_ microglobulin-creatinine ratio, *UV* urinary volume, *Ug* urinary glucose

model 1 adjusted for sex, age and BMI; model 2 plus eGFR and SUA; model 3 plus CRP, UpH, TG, UBCR, UV and Ug. Standardized beta coefficients (*St B*) refer to how many standard deviations a dependent variable will change per standard deviation increase in the predictor variable

Spearman correlation coefficient demonstrated that Cur was negatively associated with UACR (*r* = − 0.368, *P* < 0.001) before adjusting for the confounding factors. After adjusting for confounding factors in three models (model 1 adjusted for sex, age and BMI; model 2 plus eGFR and SUA; model 3 plus CRP, UpH, TG, UBCR, UV and Ug), the association of Cur with UACR was still statistically significant (*P* = 0.007; Table 4).

**Table 4:** Multiple linear regression analysis for the association of UACR (independent variable) with Cur (dependent variable)

	Model 1		Model 2		Model 3	
	*St B*	*P*	*St B*	*P*	*St B*	*P*
UACR, mg/mmol	−0.262	< 0.001	− 0.118	0.007	− 0.144	0.007
Age, y	−0.013	< 0.001	−0.175	0.003	−0.137	0.019
SUA,umol/L			−0.537	< 0.001	−0.541	< 0.001
TG, mmol/L					0.193	< 0.001
UV					0.162	0.002

*Abbreviations*: *UACR* urinary albumin to creatinine ratio, *Cur* uric acid clearance rate, *BMI* body mass index, *eGFR* estimated glomerular filtration rate, *SUA* serum uric acid, *CRP* C-reactive protein, *UpH* urinary pH, *TG* triglycerides, *UBCR* urinary β_2_ microglobulin-creatinine ratio, *UV* urinary volume, *Ug* urinary glucose

model 1 adjusted for sex, age and BMI; model 2 plus eGFR and SUA; model 3 plus CRP, UpH, TG, UBCR, UV and Ug. Standardized beta coefficients (*St B*) refer to how many standard deviations a dependent variable will change per standard deviation increase in the predictor variable

## Discussion

Our analyses showed that 24-h Uur and Cur were lower and FEur was higher in the hypertension group, stage 3–5 CKD and macro-albuminuria group than those in the normotensive group, stage 1 CKD and the normo-albuminuria group. Moreover, males had higher 24-h Uur and lower FEur than females. In addition, we found that albuminuria was negatively associated with 24-h Uur and Cur, after adjusting for multiple confounding factors.

The prevalence of hyperuricemia has been studied in general populations. In a study of 36,348 participants aged 18 years and older, Professor Liu Bicheng’s team found that between year 2009 and 2010, the corrected prevalence of hyperuricemia among Chinese adults was 8.4% [95% confidence interval (CI) 8.0–8.8%] [26]. Recently, according to a nationwide study of 22,983 adults aged≥18 years from 2007 to 2011 conducted by Peking Union Medical College Hospital, the prevalence of hyperuricemia in china was 13.0% (18.5% in men and 8.0% in women) [27]. Moreover, many studies have shown that renal underexcretion of uric acid accounts for 90% of hyperuricemia [4–6]. In the present study, we first analyzed the incidence of hyperuricemia in our 200 patients with CKD, which was 23.0%, and it increased significantly compared with the general population. In addition, among the patients with hyperuricemia, low renal excretion of uric acid accounted for 91.3%. Therefore, it indicated that whether the general population or CKD patients, insufficient renal uric acid excretion is the main reason of hyperuricemia. Additionally, the analysis showed that 24-h Uur was lower in females, which is contradictory to the previous indication that estrogen promoted urinary uric acid excretion [28]. It can be explained by the fact that most women in our study were postmenopausal. Furthermore, we found that 24-h Uur and Cur significantly reduced in patients with stage 3–5 CKD, which was consistent with the established knowledge that GFR is an important influencing factor. A large body of evidence indicated that hypertension has reciprocally influenced on or been affected by hyperuricemia [29–31], and this might support our result that hypertensive patients had lower Cur. Indeed, several investigators have reported that sodium-sensitive hypertension is associated with hypercalciuria [32]. It is highly likely that proteins responsible for renal calcium transport, including the epithelial calcium channel (TRPV5), calbindin-D28K, the Na/Ca exchanger (NCX1) and plasma membrane Ca-ATPase (PMCA), may be altered in hypertensive disease thus justifying and explaining the reported hypercalciuria. On the other hand, the study by Belge et al. [33] indicated a functional relationship between parvalbumin (PV) belonging to calbindin-D28K and the thiazide-sensitive Na + -Cl- cotransporter (NCC), the main entry step for Na + and Cl- through the apical membrane in the early distal convoluted tubule (DCT1). In vitro studies demonstrated that PV may regulate NCC expression by adjusting the Ca2 + −dependent signalling pathway [34]. Accordingly, we speculated that renal uric acid transport was also altered in hypertensive diseases. According to previous studies, high glucose levels are associated with high serum urate levels, while frank glycosuria is associated with hypouricemia [3]. Additionally, SUA concentrations may also be different according to the type of diabetes [35]. Levels of SUA are often lower in patients with type 1 diabetes compared with their nondiabetic peers [36], which may be induced by glycosuria. But whether GLUT9 is involved in this phenomenon has not been confirmed*.* In contrast, type 2 diabetes is often correlated to higher SUA concentrations [37, 38], although some studies have reported the presence of hypouricemia in type 2 diabetes [39, 40]. It is likely that the presence of insulin resistance and hyperinsulinemia stimulate the renal tubular cells to reabsorb sodium coupling with uric acid [35]. The failure to detect a change in urate excretion in diabetes in our study may be explained by the fact that increase in FEur in all the studies was related to glycosuria, not diabetes per se, which may not be prominent in a well-treated population.

Currently, the association of albuminuria with urinary uric acid excretion has not been extensively studied. Many studies focused on the association between albuminuria and SUA, and documented that SUA is positively associated with albuminuria and predicts the development of albuminuria, including general population [15], diabetes mellitus [41], and hypertension [18]. Our study revealed that UACR was negatively related to 24-h Uur and Cur, not with FEur, independent of possible confounders. The association we found is consistent with the finding in a recent study, showing that urinary uric acid excretion was reduced in proteinuria patients using HILIC-based HPLC-UV method [42]. Moreover, in another study conducted in general population, Scheven [43] found a positive association of albuminuria with tubular uric acid reabsorption, independent of potential confounders such as. hs-CRP. As to the negative association between 24-h Uur, Cur and UACR, it may well be that albumin or concomitant non-albumine compounds, such as plasmin, found in urinary of albuminuric subjects, can specifically upregulate or downregulate the expression of genes encoding for tubular uric acid transporters. This phenomenon has been shown for other, non-uric acid membrane transporters in tubular proximal epithelial cells [20–23]. However, 24-h Uur is lower at lower GFR and higher UACR. Since the patients with CKD are in steady state in these studies, this must reflect either a change in uric acid production or an increase in non-renal excretory pathways induced by metabolic changes, such as protein-energy wasting, accumulated uremic toxins, dyslipidemia and insulin resistance, in severe CKD [9, 12]. Additionally, the lower 24-h Uur and Cur can’t be explained by effects on renal transporters alone, and intestinal transport may also work. In genetic analyses of CKD cohorts, Bhatnagar et al. found that striking associations between uric acid and SNPs on ABCG2, a key transporter of uric acid by intestine, suggesting a likely compensatory intestinal extrusion of uric acid in patients with low GFR [44]. Their data supported the notion that uric acid transporters in remote organs (intestine versus kidney) may regulate serum uric acid levels, especially after acute or chronic organ damage, as suggested in the Remote Sensing and Signaling Hypothesis [45]. Accordingly, we speculated that changes of urinary uric acid excretion in the setting of CKD (low GFR and high UACR) may also be explained by the effect on intestinal transport. As far as we know, it is possible that actually uric acid changes proteinuria or that proteinuria and uric acid are dependent on the same variable. A well-designed study is needed to verify a causal relation between albuminuria and uric acid.

Interestingly, although 24-h Uur and Cur decrease with increasing CKD stage, FEur is higher. As has been previously described, with increasing severity of CKD, FEur of residual nephrons increased in order to maintain homeostasis of uric acid. Particularly when GFR had decreased to less than 15 mL/min, it increased strikingly, even to five-fold [10]. This finding may actually reflect the compensation of residual nephrons or may arise from a presently undefined influence of uremia per se. Recently, researcher Andrew Rule and colleagues have developed a method to determine GFR at the level of the single nephron, and found that CKD risk factors were associated with increased single-nephron GFR, but they thought uric acid levels were associated with lower nephron number rather than single-nephron GFR, leading to a lower total GFR [46]. Indeed, our study demonstrated that UACR was negatively associated with 24-h Uur and Cur, but not with FEur, after adjusting for confounders, suggesting that albuminuria may be associated with total uric acid excretion but was not strong enough to be correlated with the residual tubular excretion of uric acid in CKD patients. Accordingly, our findings indicate that in clinical practice, it is better to use 24-h Uur and Cur to estimate the capacity of renal uric acid excretion in CKD patients, while FEur reflects more on the compensatory residual renal tubular function and should be valued with caution in patients with reduced kidney function.

Our study has some limitations that need to be mentioned. First, the cross-sectional nature of the present study makes it hard to determine any causal relationship. Second, the effect of specific etiology of CKD was not explored, since the extent of specific renal tubular damage was different. Finally, UACR was measured once, which is known to be subject to more variability. Nonetheless, the strengths of our study included its strict exclusion criteria based on medical histories and laboratory findings, careful adjustments for possible confounders, and use of 24 h urine collection as “gold” standard method for the urinary excretion of uric acid.

## Conclusions

Influencing factors of renal uric acid excretion is complicated, and albuminuria, evaluated as UACR, was negatively correlated with 24-h Uur and Cur after adjusting for multiple confounders in Chinese CKD patients. This phenomenon may explain in part the association between albuminuria and serum uric acid. Further studies are required to determine the exact mechanism of the association of albuminuria with renal uric acid excretion.

## Abbreviations

24-h Uur: 24-h urinary uric acid excretion
ARAS: Atherosclerotic renal arterial stenosis
BMI: Body mass index
BUN: Blood urea nitrogen
CKD: Chronic kidney disease
CRP: C-reactive protein
Cur: Uric acid clearance rate
DKD: Diabetic kidney disease
eGFR: Estimated glomerular filtration rate
FBG: Fasting blood glucose
FEur: Fractional excretion of uric acid
HbA1c: Hemoglobin A1c
HDL: High-density lipoprotein
HN: Hypertensive nephropathy
LDL: Low-density lipoprotein
PGN: Primary glomerulonephritis
PKD: Polycystic kidney disease
Scr: Serum creatinine
SGN: Secondary glomerulonephritis
SUA: Serum uric acid
TC: Total cholesterol
TG: Triglycerides
TIN: Tubulointerstitial nephropathy
UACR: Urinary albumin to creatinine ratio
UBCR: Urinary β_2_ microglobulin-creatinine ratio
Ug: Urinary glucose
UpH: Urinary pH
UV: Urinary volume
